# New-Onset Type 1 Diabetes Mellitus After Treatment With Nivolumab for Melanoma

**DOI:** 10.7759/cureus.18679

**Published:** 2021-10-11

**Authors:** Sarah Baroud, Lubna Mirza

**Affiliations:** 1 Department of Internal Medicine, Sheikh Khalifa Medical City, Abu Dhabi, ARE; 2 Department of Endocrinology, Norman Regional Health System, Norman, USA

**Keywords:** melanoma, immunotherapy, nivolumab, hyperglycemia, type 1 diabetes

## Abstract

A 67-year-old female taking Nivolumab for melanoma developed a hyperglycemic emergency requiring hospital admission. She was referred to our endocrinology clinic post-discharge where she was diagnosed with new-onset Type 1 diabetes mellitus. Severe hyperglycemia and immune-mediated diabetes mellitus are extremely rare but potentially life-threatening side effects of Nivolumab.

## Introduction

Type 1 diabetes mellitus (T1DM) is an autoimmune disease that affects nearly 1.4 million adults in the United States [1]. Genetic risk factors include individuals with HLA-risk genotypes DR3/4-DQ8 or DR4/DR4 and a positive family history [2]. Non-genetic risk factors include immunization, diet, socioeconomic status, obesity, vitamin D deficiency, perinatal factors, and a history of viral infections, in particular enterovirus infections. Traditional studies have alluded the cause of T1DM to the immune system inappropriately producing antibodies against its own pancreatic β-cells, the prime source of insulin production [3]. However, newer studies have shown that a fault in the gene sequence of β-cells, which is predominantly seen following immunotherapy treatment, may be the underlying cause behind the inappropriate activation of the immune system [4].

The autoimmune destruction of pancreatic β-cells results in a rise in blood glucose levels due to the depletion of insulin, an essential protein regulator of blood glucose [5]. An individual may remain asymptomatic until an incidental discovery is made during routine screening [3], or they may manifest clinically with polyuria, polydipsia, weight loss, fatigue, dyspnea, vomiting, abdominal pain, polyphagia, somnolence, lethargy, and even coma. Diabetic ketoacidosis is a life-threatening medical emergency that is most often the first presentation of T1DM [6].

Nivolumab is a human immunoglobulin G4 (IgG4) monoclonal antibody used in the treatment of colorectal cancer, head and neck cancer, hepatocellular carcinoma, melanoma, renal cell cancer, lung cancer, urothelial carcinoma, and Hodgkin’s lymphoma. It works by blocking programmed death 1 (PD-1) receptor, an immune system checkpoint and cell surface protein found on activated T-cells, from binding to programmed death ligand 1 (PD-L1). This disruption in the PD-1/PD-L1 axis, which is responsible for modulating T-cell activity and critical in tumorigenesis, restimulates the immune system’s anti-neoplastic activity, resulting in a decrease in tumor growth and consequently, an increase in autoimmune-associated side effects. Severe hyperglycemia and immune-mediated diabetes mellitus secondary to Nivolumab are rare side effects occurring in less than ≤1% of patients with a median time to onset of 12 weeks (range: 5-34 weeks) from the start of treatment [7, 8].

In this case report, we discuss the case of a 67-year-old female who presented to the hospital with hyperglycemia-related signs and symptoms six weeks after initiating Nivolumab immunotherapy for melanoma. Predominant findings included polyuria, polydipsia, dehydration, fatigue, and an elevated blood glucose level, and she was admitted for a life-threatening hyperglycemic emergency. The patient was treated and subsequently discharged, and was referred to our endocrinology clinic for further workup where a diagnosis of new-onset T1DM was made.

## Case presentation

A 67-year-old Caucasian woman was referred to our clinic for newly diagnosed hyperglycemia. For the past year, she had been following up with us for her thyroid disease. She has had Hashimoto's Thyroiditis for the last 15 years and has been controlling it with Levothyroxine and Liothyronine Sodium. Her medical history was also remarkable for a melanoma on her left forearm that was treated surgically 5 years prior. Axillary sentinel lymph nodes had been removed at the time and showed no metastases. No chemotherapy or radiation was required and regular follow-up with her dermatologist has shown no signs of recurrence since. Her family history is negative for thyroid cancer, functional thyroid disease, diabetes, or autoimmune diseases. During her last visit, which was 2 months before her admission to the hospital, her labs showed a normal blood glucose level (Table 1).

**Table 1:** Laboratory investigations at our clinic 2 months before hospital admission ALT: alanine transaminase; ALP: alkaline phosphatase; AST: aspartate aminotransferase; GFR: glomerular filtration rate; TSH: Thyroid-stimulating Hormone; T4: Thyroxine; T3: Triiodothyronine; dl: deciliter; fl: femtoliters; g: grams; IU: international unit; L: liter; mEq: milliequivalent; mg: milligram; mIU: milli-international units; ml: milliliter; mmol: millimole;  ng: nanograms; pg: picograms; U: Units

Lab Marker	Finding	Reference
White Blood Count	5.4	4.5-11.0 10*9/L
Red Blood Count	4.80	4.00-5.20 10*12/L
Hemoglobin	15.2 g/dl	12.0-16.0 g/dl
Hematocrit	43.6 %	36.0-46.0 %
Mean Corpuscular Volume	90.8	80.0-100.0 fl
Platelet Count	313	150-400 10*9/L
Sodium	137 mmol/L	136-144 mmol/L
Potassium	4.7 mmol/L	3.4-5.1 mmol/L
Calcium	10.4 mg/dl	8.4-10.0 mg/dl
Chloride	105 mmol/L	100-109 mmol/L
Carbon Dioxide	25 mmol/L	24-32 mmol/L
Anion Gap	12 mEq/L	9-14 mEq/L
Blood Urea Nitrogen	19 mg/dl	7-20 mg/dl
Creatinine	0.83 mg/dl	0.60-1.20 mg/dl
GFR, Estimated	> 60 ml/min	>60 ml/min
Glucose	93 mg/dl	65-100 mg/dl
Bilirubin Total	0.4 mg/dl	0.3-1.4 mg/dl
AST	17 IU/L	15-44 IU/L
ALT	21 IU/L	15-44 - IU/L
Total Protein	7.2 g/dl	6.1-7.9 g/dl
Albumin	4.5 g/dl	3.5-5.1 g/dl
ALP	95 U/L	41-126 U/L
TSH	1.79 mIU/L	0.49 mIU/L - 4.00 mIU/L
Free T3	3.61 pg/ml	2.3 pg/ml - 4.4 pg/ml
Free T4	1.01 ng/dl	0.60 ng/dl - 2.00 ng/dl

A medical examination was only notable for a BMI of 29 kg/m2 (normal reference range: 18.5 to 24.9 kg/m2); she had no clinical signs of thyromegaly, lymphadenopathy, acanthosis nigricans, or vitiligo. We had asked her to follow-up with us in the next 6 months.

During this time, she had consulted her dermatologist for a melanoma on her chin. The melanoma on her chin was removed surgically and she was advised to start immunotherapy with Nivolumab once every two weeks. During her sixth week of treatment, she was admitted to the hospital with fatigue, dry mouth, excessive thirst, and excessive urination. Laboratory findings at the time of presentation are provided below (Table 2) and were consistent with a diagnosis of diabetic ketoacidosis (DKA). 

**Table 2:** Laboratory investigations at time of hospital presentation POC: Point of Care ; VBG: Venous Blood Gas; dl: deciliter; L: liter; mEq: milliequivalent; mg: milligram; mmhg: millimeters of mercury; mmol: millimole

Lab Marker	Finding	Reference
POC Sodium	137 mmol/L	136-144 mmol/L
POC Potassium	6.7 mmol/L	3.4-5.1 mmol/L
POC Chloride	111 mmol/L	100-109 mmol/L
POC Magnesium	2.9 mmol/L	0.85 -1.10 mmol/L
POC Ionized Calcium	1.25 mmol/L	1.15 -1.30 mmol/L
POC Anion Gap	25 mEq/L	9-14 mEq/L
POC Blood Urea Nitrogen	43 mg/dl	7-20 mg/dl
POC Creatinine	1.2 mg/dl	0.60-1.20 mg/dl
POC Lactic acid	2.20 mmol/L	0.5 - 2.2 mmol/L
POC Glucose	>700 mg/dl	65-100 mg/dl
Urine PH	5.0	4.6 - 8.0
Urine specific gravity	1.021	1.005 - 1.030
Urine Ketone	80 mg/dL	Small: <20mg/dL , Moderate: 30 to 40 mg/dL, Large: >80 mg/dL
Urine Protein	30 mg/dL	0 - 14 mg/dL
Urine Glucose	500> mg/dl	0 -15 mg/dl
POC VBG PH	7.025	7.31-7.41
POC VBG pCO2	25.4 mmhg	41 – 51 mmhg
POC VBG pO2	45 mmhg	30 -40 mmhg
POC VBG HCO3	6.6 mmol/L	23-29 mmol/L
POC Base Excess	-24 mEq/L	−2 to +2 mEq/L

The patient was discharged on Insulin Glargine after stabilization and referred to our endocrinology clinic for further evaluation. On presentation, she had normal vital signs and physical examination was unremarkable except for a BMI of 27.86 kg/m2 (normal reference range: 18.5 to 24.9 kg/m2). Laboratory investigations were ordered, and the results were consistent with a new diagnosis of Type 1 diabetes mellitus (Table 3). 

**Table 3:** Laboratory investigations following discharge from the hospital GAD65: Glutamic acid decarboxylase; mg: milligram; dl: deciliter; IU: international unit; ml: milliliter; ng: nanograms

Lab Marker	Finding	Reference
Random Blood Sugar	271 mg/dl	65 mg/dl - 100 mg/dl
Hemoglobin A1C	7.0%	5.7% -6.4%
GAD65 Antibodies Level	250.0 IU/ml >	0.0 IU/ml - 5.0 IU/ml
C-Peptide Level	< 0.05 ng/ml	0.90 ng/mL-4.00 ng/mL

Nivolumab treatment was discontinued, and 10 units of subcutaneous Novolog (Insulin Aspart) which was to be taken three times a day (TID) was added to her current medication regimen. She was counseled on her condition and advised to follow-up with the diabetes education team.

## Discussion

Type 1 diabetes mellitus (T1DM) commonly occurs in children, with one in every four cases diagnosed in adults [3]. Various genetic and environmental factors contribute to the pathogenesis of this chronic condition. Autoimmunity is the underlying reason behind the destruction of the pancreatic β-cells and the depletion of its byproduct, insulin, which is a necessity in blood glucose homeostasis [5]. Recent studies now demonstrate that a fault in the β-cell gene sequence is behind the autoimmune destruction of the pancreas. This phenomenon, which was best observed in cancer patients who had received immunotherapy treatment, revealed that previously functioning β-cells began to produce non-functioning proteins, a feature commonly seen in cancer cells. This in turn prompts the normal antitumor response of the immune system against these pancreatic cells and therefore may explain why certain cancer patients develop T1DM after receiving immunotherapy treatment [4].

Nivolumab is an Immunoglobulin G4 (IgG4) monoclonal antibody used in the treatment of various malignancies that works by blocking the activated T-cell PD-1/PD-L1 axis to suppress cell proliferation and neoplastic activation. Collectively, immunotherapy medications are called immune checkpoint inhibitors (ICI) and they all carry the risk of immune-related adverse events (irAE). The risk of severe hyperglycemia and immune-mediated diabetes mellitus with Nivolumab is ≤1% [7, 8].

T1DM is diagnosed based on clinical and laboratory findings. In our patient, a sustained elevated blood sugar level and positive autoantibodies confirmed the diagnosis [3]. T1DM carries the risk of macrovascular and microvascular complications such as diabetic nephropathy, retinopathy, and cardiovascular disease [9]. It also carries the risk of DKA, a medical emergency, and the most common initial presentation in newly diagnosed T1DM [6]. Treatment is lifelong with tight glycemic control and insulin replacement [10]. As the frequency of ICI use increases, so does the frequency of irAE. Currently, there is a 10-20% risk of irAE in all patients using ICI [8]. This brings up the importance of discussing the risk of developing severe hyperglycemia and immune-mediated diabetes mellitus with Nivolumab immunotherapy and the significance of early recognition and intervention to prevent long-term complications.

## Conclusions

The risk of developing severe hyperglycemia and immune-mediated diabetes mellitus in cancer patients receiving Nivolumab immunotherapy is ≤1%. However, with the increasing use of immune checkpoint inhibitors (ICI), the risk of immune-related adverse events (irAE) also increases. Moreover, ICI may accelerate a pre-existing autoimmune process in patients with other autoimmune diseases or patients with a high propensity for developing autoimmune diseases, such as those with risk factors or a positive family history, thereby further increasing the risk of irAE. Therefore, it is important for physicians to be aware of these risks to be able to intervene early and reduce associated morbidity and mortality. 

## References

[1] (2021). National Diabetes Statistics Report, 2020. https://www.cdc.gov/diabetes/library/features/diabetes-stat-report.html.

[2] Steck AK, Rewers MJ (2011). Genetics of type 1 diabetes. Clin Chem.

[3] Levitsky L.L, & Misra M. (2021). Epidemiology, presentation, and diagnosis of type 1 diabetes mellitus in children and adolescents. https://www.uptodate.com/contents/epidemiology-presentation-and-diagnosis-of-type-1-diabetes-mellitus-in-children-and-adolescents.

[4] City of Hope. (2017 (2021). New potential cause of type 1 diabetes. http://www.sciencedaily.com/releases/2017/03/170301085412.htm.

[5] Ozougwu J, Obimba K, Belonwu C, Unakalamba C (2013). The pathogenesis and pathophysiology of type 1 and type 2 diabetes mellitus. J Physiol Pathophysiol.

[6] Westerberg DP (2013). Diabetic ketoacidosis: evaluation and treatment. Am Fam Physician.

[7] Westerberg Westerberg, D.P. D.P. (2021). Drug Name: Nivolumab. American Family Physician.

[8] Godwin JL, Jaggi S, Sirisena I, Sharda P, Rao AD, Mehra R, Veloski C (2017). Nivolumab-induced autoimmune diabetes mellitus presenting as diabetic ketoacidosis in a patient with metastatic lung cancer. J Immunother Cancer.

[9] Mahajan R (2015). Blood test to help diagnose type 1 diabetes approved by Food and Drug Administration. Int J Appl Basic Med Res.

[10] McCulloch McCulloch, D. K. (2018 (2021). Management of blood glucose in adults with type 1 diabetes mellitus. https://www.uptodate.com/contents/management-of-blood-glucose-in-adults-with-type-1-diabetes-mellitus.

